# Volumetric evaluation of pharyngeal segments in obstructive sleep apnea patients^☆^^☆☆^

**DOI:** 10.1016/j.bjorl.2016.12.001

**Published:** 2017-01-30

**Authors:** Marcos Marques Rodrigues, Valfrido Antonio Pereira Filho, Mário Francisco Real Gabrielli, Talles Fernando Medeiros de Oliveira, Júlio Américo Pereira Batatinha, Luis Augusto Passeri

**Affiliations:** a Universidade de Araraquara, Faculdade de Medicina da Araraquara, Divisão de Otorrinolaringologia, Araraquara, SP, Brazil; b Universidade Estadual Paulista “Júlio de Mesquita Filho” (UNESP), Faculdade de Odontologia de Araraquara, Departamento de Diagnóstico e Cirurgia, Programa de Cirurgia Oral e Maxilofacial, Araraquara, SP, Brazil; c Universidade Estadual Paulista “Júlio de Mesquita Filho” (UNESP), Faculdade de Odontologia de Araraquara, Departamento de Ortodontia, Araraquara, SP, Brazil; d Universidade de São Paulo (USP), Faculdade de Medicina, São Paulo, SP, Brazil; e Universidade Estadual de Campinas (UNICAMP), Faculdade de Medicina e Ciências, Departamento de Cirurgia, Cirurgia Oral e Maxilofacial, Campinas, SP, Brazil

**Keywords:** Upper airway, Obstructive sleep apnea, Cone beam CT, Via aérea superior, Apneia obstrutiva do sono, TC de feixe cônico

## Abstract

**Introduction:**

Obstructive sleep apnea occurs by recurrent collapse of the upper airway during sleep, resulting in total (apnea) or partial (hypopnea) reduction of the airflow and has intimate relation with changes in the upper airway. Cone Beam CT allows the analysis of the upper airway and its volume by three-dimensional reconstruction.

**Objective:**

To evaluate a possible correlation between the volume of the upper airway and the severity of the obstructive sleep apnea.

**Methods:**

A retrospective study was performed reviewing polysomnographic data and Cone Beam CT records of 29 patients (13 males and 16 females). The correlation between the volume of the nasopharynx, the oropharynx and the total superior pharynx with the AHI was assessed by Pearson's rank correlation coefficient.

**Results:**

The obstructive sleep apnea severity division was: ten patients had severe, 7 had moderate, 6 had mild and 6 of them were healthy. The correlation between the nasopharynx, the oropharynx and the total superior pharynx volumes and the Apnea-Hypopnea-Index was respectively: −0.415 (*p* = 0.025), 0.186 (*p* = 0.334) and −0329 (*p* = 0.089). The Spearman's rank controlled by the Body Mass Index, the age and the gender was: −0.206 (*p* = 0.304), −0.155 (*p* = 0.439) and 0.242 (*p* = 0.284).

**Conclusion:**

There is no correlation between the volume of the airway and the obstructive sleep apnea, assessed by Apnea-Hypopnea-Index and controlled by the Body Mass Index, the age and the gender. The volume of the upper airways as an isolated parameter did not correlate to the severity of the obstructive sleep apnea syndrome, and should be evaluated together with other factors.

## Resumo

**Introdução:**

A Apneia Obstrutiva do Sono ocorre por colapso recorrente das vias aéreas superiores durante o sono, resultando em redução total (apneia) ou parcial (hipopneia) do fluxo aéreo, tendo relação estreita com alterações nas vias aéreas superiores. A TC de feixe cônico permite a análise da via aérea superior e seu volume através da reconstrução tridimensional.

**Objetivo:**

Avaliar uma possível correlação entre o volume da via aérea superior e a gravidade da apneia obstrutiva do sono.

**Método:**

Realizou-se um estudo retrospectivo, com revisão de dados polissonográficos e registros de TC de feixe cônico de 29 pacientes (13 do sexo masculino e 16 do sexo feminino). A correlação entre o volume total da nasofaringe, a orofaringe e a faringe superior com o IAH (Índice de Apneia-Hipopneia) foi avaliada pelo coeficiente de correlação de Pearson.

**Resultados:**

A divisão por gravidade da Apneia Obstrutiva do Sono foi: dez pacientes apresentaram apneia na forma severa, 7 apresentaram apneia moderada, 6 tinham a forma leve e 6 estavam saudáveis. A correlação entre a nasofaringe, a orofaringe e os volumes da faringe superior e o Índice de Apneia-Hipopneia foram respectivamente: −0,415 (*p* = 0,025), 0,186 (*p* = 0,334) e −0329 (*p* = 0,089). A classificação de Spearman controlada pelo Índice de Massa Corporal, idade e sexo foi: −0,206 (*p* = 0,304), −0,155 (*p* = 0,439) e 0,242 (*p* = 0,284).

**Conclusão:**

Não há correlação entre o volume da via aérea e a apneia obstrutiva do sono, avaliada pelo índice de apneia-hipopneia e controlada pelo índice de massa corporal, idade e sexo. O volume das vias aéreas superiores como parâmetro isolado não se correlacionou com a gravidade da síndrome da apneia obstrutiva do sono, e deve ser avaliado em conjunto com outros fatores.

## Introduction

Obstructive sleep apnea (OSA) is the main sleep respiratory disorder.^1^ OSA is defined as a recurrent collapse of the upper airway during sleep, resulting in a total (apnea) or partial (hypopnea) reduction of the airflow.^2^ Clinical findings include increased neck circumference, nasal obstruction, turbinate hypertrophy, septum abnormalities, flaccid palate, pharyngeal tonsils hypertrophy, macroglossia and oropharyngeal obstruction.^3^ The risk factors associated with the apnea onset include male gender, Body Mass Index (BMI) > 25 kg/m^2^, low socioeconomic status, advanced age and menopause.^4^ The airway patency is also reported as a determinant factor of the OSA. Obesity, edema and genetic factors contribute its onset; as such situations may promote variations in the airway volume.^3^ OSA prevalence in Western populations estimated that from 1% to 5% of the adults have OSA syndrome.^1^

The airway study in OSA patients has had an important advancement due to the use of the Cone-Beam Computed Tomography (CBCT), in association with 3D reconstruction software. That allows tridimensional airway evaluation, airway volume determination and detection of sites of maximum constriction.^5^ These parameters are very important in the OSA evaluation. This is a disease that affects primarily the upper airway and then induces cardiovascular and metabolic alterations. The 3D airway evaluation can leave us to determine the different sites of obstruction and program a correct treatment of the airway patency. The 2D evaluation permits only measurement in one plan and may lead to misleading interpretations of the upper airway structure.^5^

Studies correlating the OSA with the upper airway volume and tomographic abnormalities are rare and conflicting. The CBCT is a powerful tool on the OSA understanding and should be explored to facilitate the planning for the therapy of OSA patients.^5^

The aim of this study is to search for a correlation between the volume of the pharynx assessed by CBCT and the severity of OSA.

## Methods

This is a retrospective study reviewing the medical records of 33 patients. The study was approved by the Ethics and Research Committee (registration 13185113.9.0000).

The inclusion criteria were minimum age of 18 years, individuals who had clinical evaluation for sleep respiratory disorders associated to signs and symptoms, such as snoring, daytime sleepiness, witnessed apneas and choking during sleep. Patients with morbid obesity (BMI > 40), craniofacial abnormalities, nasal obstruction by polyposis, craniofacial or airway tumor; larynx or pharynx paralysis and previous maxillofacial or upper airway surgery were excluded. The study was limited to patients with sufficient data on demographics, BMI, basal polysomnography, and adequate CBCT.

The sample size was calculated using sample error by 7%, confidence level of 90% and considering an airway volume difference of 6% between of OSA Severity groups. The sample population was 1920 patients, which is the outpatient service capacity during the analysis period of this study. The sample size was calculated in 29 subjects.

To obtain the tomographic images patients were seated, the main position of physical examination, in natural head position, and were instructed not to swallow during the exam. The images were obtained in a CBCT scanner (I-Cat, KaVo – Brazil) set up at 120 kVp, 36 mA, 0.25 mm voxel and FOV of 16 × 22 cm, from the vertex of the cranium to C3 level. All images were stored on a DVD for specific software analysis. The dicom images were imported and reconstructed using the Dolphin software (Dolphin Imaging Management Solutions, Chatsworth, CA, USA). The orientation of each dataset was standardized according to the Frankfurt horizontal plane and mid-sagittal plane using volume rendering and multiplanar views, as described by Guijarro-Martinez and Swennen.^6^ The volumetric analysis of the tridimensional pharynx's subregions was done by this same software, summing the volumes in cubic millimeters.

To measure the volumes, the sagittal slices were selected and the planes were formed resorting to anatomical references, thus defining its upper and lower limits for the determination of the volume. The pharynx was segmented in nasopharyhnx and oropharynx. The limits of each portion were determined as used in the validation study of Guijarro-Martinez and Swennen.^6^ The limits of the nasopharynx were anteriorly a plane perpendicular to the Frankfort Horizontal Plane (FHP) passing through the Posterior Nasal Spine (PNS), and inferiorly a plane parallel to FHP passing through the PNS and extended to the posterior wall of the pharynx. The oropharynx started at this last plane and its inferior limit was a plane parallel to FHP passing through the most anteroinferior point of the body of C3. The volume of the Total Superior Pharynx was obtained by the sum of the Nasopharynx and the Oropharynx volumes.

The polysomnographies were performed after obtaining the CBCT. The sleep was evaluated during an average period of 6 h. The electrophysiological variables evaluated during sleep were: electroencephalogram (EEG), electrooculogram (EOG), electromyogram (EMG), electrocardiogram (EKG), airway flow (oral and nasal), respiratory effort (thoracic and abdominal), other body movements (measured by EMG), blood gases (oxygen saturation) and body temperature. The exams were evaluated by criteria of 2012 AASM Manual.^7^ A sleep disorders specialized physician (M.M.R.) obtained the apnea/hypopnea index by summing the apnea and hypopnea events divided by hours of sleep. According to the results, OSA was classified as absent (AHI < 5 events/h), mild (5 AHI < 15 events/h), moderate (15 ≤ AHI < 30 events/h) or severe (AHI ≥ 30 events/h).

The measurements of the 3D tomographic images were obtained by a calibrated examiner who was blind to any other study data, such as anthropometric elements, physical examination and polysomnography. Two volume determinations were obtained separated by a 30 day interval, and a mean of them was used. The reproductibility was evaluated by the Dahlberg's formula (and the Intraclass Correlation Coefficient [ICC]). The Data were analyzed by statistical descriptive tests and the frequency of the results. The Pearson rank correlation coefficient was used for the correlation of the pharynx's subregions volume and the AHI. The SAS System for Windows (Statistical Analysis System), version 9.3 software (SAS Institute Inc, 2002–2008, Cary, NY, USA) was used for the analysis with a significance level of 5% (*α* = 0.05).

## Results

Thirty-three patients were evaluated between June 2012 and December 2013. Four patients were excluded. Two of them presented inadequate tomographic exams and 2 had incomplete records. Therefore, 29 patients were included in the study, 16 female (55%) and 13 male (45%).

The descriptive data of anthropometric variables in each OSA severity group can be found in Table 1. The Kruskal–Wallis *H*-test was used to evaluate the equality of these groups. Considering age and gender groups were similar. Groups of OSA severity were different in BMI only. Table 2 demonstrates that variables had a normal distribution by Kolgomorov–Smirnov test. A paired Marginal Homogeneity test was used to evaluate the strength of the airway volume measurement. The results of the reproductibility analysis are summarized in Table 3.

**Table 1:** Description of OSA severity according to AHI.

OSA severity	Frequency	Percent	BMI mean	Age mean	Male percent
Normal	6	20.7%	25.85	50.50	50%
Mild	6	20.7%	30.01	51.00	0%
Moderate	7	24.1%	30.72	46.85	57%
Severe	10	34.5%	31.77	40.00	60%
Kruskal–Wallis *H*-test	29	100%	0.014 (s)	0.188 (ns)	0.108 (ns)

**Table 2:** Description and normality test of continuous variables.

Variable	Mean	Kolgomorov–Smirnov test
BMI (kg/m^2^)	29.72	0.957
Age (years)	46.10	0.870
AIH (ev/h)	27.27	0.216
Nasopharynx volume (mm^3^)	5467.67	0.958
Oropharynx volume (mm^3^)	9953.71	0.854
Total superior pharynx volume (mm^3^)	15,421.39	0.921

BMI, Body Mass Index; AIH, Apnea-Hypopnea Index.

**Table 3:** Marginal Homogeneity test, Dahlberg's error and Intraclass Correlation Coefficient (ICC) for the two volume determinations in mm^3^.

	Mean	*p*	Dahlberg's error	ICC
Nasopharynx volume	5443.12	0.281	173.47	0.988
Nasopharynx volume^a^	5492.23			
Oropharynx volume	9985.96	0.125	160.29	0.997
Oropharynx volume^a^	9921.45			
Total superior pharynx volume	15,429.08	0.654	132.14	0.999
Total superior pharynx volume^a^	15,413.69			

a Second volume determination.

The AHI was statistically evaluated as a continuous variable, the Spearman's rank correlation coefficient was chosen. This analysis results appear in Table 4, Table 5. In Table 5, the Spearman's rank correlation was controlled by the BMI, the age and the gender. There was a statistically significant correlation between AIH and BMI. There was a moderate correlation between AIH and Nasopharynx. The relationship between Oropharynx and Total Superior Pharynx volume measurements with AHI was low and not statistically significant. The correlation between the AIH and pharynx subregions volume was low and was not statistically significant when controlled by the BMI, the age and the gender.

**Table 4:** Spearman's rank correlation coefficient between AHI anthropometric variables.

	AHI	BMI	Age	Gender
Pearson correlation	1	0.570^a^	−0.355	−0.182
Sig. (2-tailed)		0.002	0.059	0.344
*n*	29	29	29	29

a Correlation is significant at the 0.01 level (2-tailed).

**Table 5:** Spearman's rank correlation coefficient between AHI and volume or pharynx segments.

	AHI	Nasopharynx volume	Oropharynx volume	Total superior pharynx volume
Pearson correlation	1	−0.415^a^	−0.186	−0.329
Sig. (2-tailed)		0.025	0.334	0.089
*n*	29	29	29	29

a Correlation is significant at the 0.05 level (2-tailed).

## Discussion

OSA is a dynamic disease, which develops under upper airway total or partial obstruction during sleep. Patients can show one or more obstructive sites located on nasal cavity, oropharynx, base of tongue and hypopharynx.^8^ The whole airway evaluation is fundamental on diagnosing OSA. Surgical treatment efficacy relies on determining and handling all multiple obstructive sites of the upper airway.^8^

In this study all OSA severity patient groups were studied, accordingly to Table 1. The higher incidence of severe patients was considered normal, since the patients came from an OSA reference center. The anthropometric data obtained, such as mean BMI compatible with obesity and prevalent age between 40 and 50 years allows to conclude that OSA predominated in obese middle aged individuals in this study.^4^ Obesity is an important variable in OSA evaluation. In this sample OSA severity groups were statistically similar in age and gender, but different in BMI (Table 4).

The correlation between the pharynx segments volume and OSA (Table 5), which was evaluated by the AHI was moderate and negative in relation to the Nasopharynx (−0.437, *p* = 0.018), but this relationship was not maintained when it was controlled by the BMI, the age and the gender (Table 6). There was no correlation between the oropharynx and the total superior pharynx volume and the AHI (Table 5) and the BMI did not influence this relationship (Table 6). This data goes against the common sense, where it is believed that surgical enlargement of the pharynx is the primary intervention in the airway treatment in patients with OSA.^8^

**Table 6:** Spearman's rank correlation coefficient between volume and AHI, controlling by BMI, age and gender.

	Nasopharynx volume	Oropharynx volume	Total superior pharynx volume	AHI
Correlation	−0.206	−0.155	−0.242	1.000
Sig (2-tailed)	0.304	0.439	0.284	

There are few studies about the relationship between nasopharynx and OSA in adults. Cai et al., 2010^9^ found that the narrowing of the upper airway in OSA patients can be mainly attributed to the nasopharynx, but they considered the influence of hard palate length in this relationship. It is probable that BMI is more important in the evaluation of OSA than nasopharynx as isolated variable, because this subregion volume did not remain statistically significant when considering BMI.

Abramson et al., 2010,^10^ have shown that the linear analysis of the airway volume and the AIH was not positive. OSA is also related to the airway length. The more the length increases, the higher will be the chances of collapse to occur. Since the airway is a simple conduit, the airflow resistance is combined serially resulting in an increase of the total resistance.^10^ The volume analysis controlling BMI and age also did not show any interaction with OSA.

Similar results were found in a South Korean study, where patients were divided into two groups with AHI higher or lower than 30 events/h, but no difference was found between the airway volume of the two groups.^11^ Another study comparing tomographic airway abnormalities in OSA patients with open or closed mouth found a significant reduction of upper airway length when the mouth was open. On the airway volume evaluation there was no difference between the two groups. Therefore, the airway volume did not significantly fluctuate in different OSA severity patients or due to mouth opening.^12^

These studies evaluated the relationship between OSA and the total airway volume. In our sample the pharynx was studied by his subregions, assessing the affinity between OSA and pharynx segments (nasopharynx and oropharynx) separately.

The volume of pharynx, as assessed in this study, does not predict the severity of OSA. The disease is multifactorial and the evaluation of a localized sector of the airway may lead to misinterpretation and treatment failure because it does not assess all levels of the airway and does not evaluate the factors of extrinsic compression of the pharynx.^13^, ^14^ It is important to considerate that the volume of pharynx is not a static but a dynamic measure. It is affected by swallowing, breathing, and positioning. In this study, the pharynx volume was assessed by still pictures. The volume of the pharynx may vary in the same individual, although all patients have received the same instructions at the time of the image acquisition.

The American Academy of Sleep Medicine (AASM) published in 2010 a meta-analysis on surgical procedures of the upper airway. This study concluded that procedures in an isolated region of the airway showed no consistency in the reduction of AHI, with residual OSA after the procedure. The best results were obtained with multilevel surgery approach.^14^ It is inferred that one of the causes of these findings is the lack of correlation between the volume of airway with AHI.

OSA is a disease related to intrinsic and extrinsic changes of the entire airway. Despite the fact that the pharynx plays a central role in the development of the disease, there was no correlation between the volume of this isolated sector of the airway and OSA severity. CBCT is a fast and low risk method for patient examination, with small emission of radiation. It reaches up to three times the speed of those used in conventional CT while emitting 10 times less radiation to the patients. The cycle lasts no more than 40 s and the emitted beam is pulsatile, reducing the amount of radiation employed.

However, it is a static test, done with the patient seated. The measurement of AHI, which defines the severity of OSA, is obtained with the patient lying down in natural sleep. The difference of position and the static analysis of the pharynx can lead to different results between AHI and volume. It is important to note that in current practice the patients are examined in a wakefulness state and in a seated position. Surgical decisions taken at this position can leave a misinterpretation of pharynx spaces, since the dimensions of the oropharynx do not relate with the severity of OSA in our sample.

## Conclusion

In the population studied, considering the Body Mass Index, the volume of the subregions of the pharynx has no linear relationship with the severity of the obstructive sleep apnea, as measured by the Apnea-Hypopnea Index. The Nasopharynx volume had a negative and a significant relationship with the obstructive sleep apnea severity but this nexus was not sustained when the BMI, the age and the gender were considered.

## Conflicts of interest

The authors declare no conflicts of interest.
